# A Centralized Outbreak of Varicella among Children Attending Preschool in Suzhou, China

**DOI:** 10.1155/2020/6183936

**Published:** 2020-05-02

**Authors:** Min Zhang, Guo-Ping Gui, Feng Guo, Xin-Fang Fan, Ri-Sheng Zha

**Affiliations:** Suzhou National New and Hi-tech Industrial Development Zone Center for Disease Control and Prevention, Suzhou, Jiangsu 215011, China

## Abstract

**Background:**

Varicella vaccine is available for voluntary purchase with a single dose currently recommended for children aged ≥12 months. An epidemiological study was undertaken in order to determine the characteristics of the outbreak, assess vaccine effectiveness, and examine risk factors for vaccine failure.

**Methods:**

A varicella case was defined as a generalized papulovesicular rash (without other apparent causes) in a child without prior varicella attending the kindergarten during February 22 to April 7 of 2016. Varicella among vaccinated children (breakthrough varicella) was defined as varicella occurring >42 days after vaccination. Children's vaccination status was verified with immunization records through local vaccination information platform.

**Results:**

Of the 738 children, 664 (90.0%) had no prior varicella history. Of these, 364 (54.8%) had received a single-dose varicella vaccine before outbreak. A total of 30 cases occurred in the outbreak, and 9 of them (30%) had breakthrough varicella. Age at vaccination (<15 months vs. ≥15 months) and time since vaccination before the outbreak (<3 years vs. ≥3 years) were not related to the occurrence of breakthrough varicella (*P* > 0.05). Single-dose varicella vaccination was 64.7% effective in preventing any varicella.

**Conclusions:**

Single-dose varicella vaccine is effective in reducing the varicella attack rate, but not high enough to prevent outbreak. Timely detection and effective isolation are key factors in controlling varicella. Improving single-dose vaccination coverage and implementing two-dose vaccination strategy should be recommended to provide excellent protection to prevent varicella in the future in Suzhou.

## 1. Introduction

Varicella is a highly contagious disease caused by varicella-zoster virus and spreads from person to person by direct contact or through the air by aerosols from infected persons [1, 2]. Despite the fact that varicella is usually self-limiting and lasts within 5-10 days, infection can lead to severe complications and occasional fatalities, particularly in infants and immunocompromised persons [2].

Varicella is one of the most common childhood diseases, with the highest incidence occurring among children aged 1-6 years [2]. The outbreak of varicella is particularly common in preschools and schools and can last several months, causing much disruption [3]. A live attenuated varicella vaccine was developed in 1974 and licensed for use in China in 1998 [4, 5]; since then, the vaccine was wide-spread, and it has been identified to be safe and effective. Moreover, the dramatic decline in varicella disease after the introduction of the vaccine also implied the vaccine's high effectiveness in the prevention of varicella disease [6, 7]. Currently, varicella vaccine is available for voluntary purchase but not included in the national or municipal childhood immunization programs in China. Although great achievement had been made in reducing varicella incidence, outbreaks continued to be reported, especially in preschools, schools, etc. [8–10].

In March 2016, Suzhou National New and Hi-tech Industrial Development Zone (SND) Center for Disease Control and Prevention (CDC) was notified of a centralized outbreak in a preschool for children aged 3-6 years in SND. SNDCDC subsequently undertook an investigation to describe the outbreak and identify challenges in case management and outbreak control in this setting.

## 2. Methods

### 2.1. Outbreak Setting

From March 2016 to April 2016, an outbreak occurred in a public preschool located in a community of Suzhou, China. During the outbreak, there were 738 children aged 3-6 years enrolled in the preschool. The preschool consisted of 20 classes, including 9 bottom classes, 5 middle classes, and 6 top classes.

### 2.2. Case Definition

All varicella cases were differentially diagnosed by clinical physicians in local hospitals on the basis of the symptoms of specific papulovesicular rash without other apparent causes and fever and exposure to varicella. All varicella cases in the outbreak, occurring between March 3 and April 5, 2016, in the preschool, were identified and collected if the following were met: (1) cases diagnosed by a physician, (2) hospital medical records, and (3) affirmative answer in the questionnaire for the following item: “Has your child gotten varicella infection during the outbreak?” For all cases, their parents were interviewed by telephone to further confirm the case status. Breakthrough disease was defined as varicella disease in a child who had been vaccinated at least 42 days before papulovesicular rash onset. The study protocol was approved by the Ethics Committee of SNDCDC.

### 2.3. Epidemiological Investigation

Self-designed questionnaires were distributed to parents of all children to collect data on demographics, varicella disease history, and vaccination disease status, including dates of vaccination. Varicella vaccination history was verified through immunization records from the management system of expanded program on immunization (EPI). Information of clinical presentations was obtained from parents of all varicella and breakthrough disease cases by telephone. All cases' medical records were also collected from the related hospitals. Detailed records of absence, which were collected by the preschool, were used to trace the cause of outbreak.

### 2.4. Vaccine Effectiveness (VE)

The attack rates in unvaccinated children (ARU) and vaccinated children (ARV) were calculated, respectively. VE was calculated as VE = (ARU − ARV)/ARU × 100%. Children with prior history of varicella before the outbreak, vaccinated less than 42 days before disease onset and the index case, were excluded from VE analysis.

### 2.5. Statistical Analysis

Data were entered into Epi Info (version 7.1.5.2, Centers for Disease Control and Prevention). Pearson's chi-square test and Fisher's exact test were used for the comparison of proportions. All the statistical analyses were performed by SPSS 13.0 software. A two-sided *P* value < 0.05 was regarded as statistically significant.

## 3. Results

### 3.1. Study Population Characteristics

**Figure 1:**
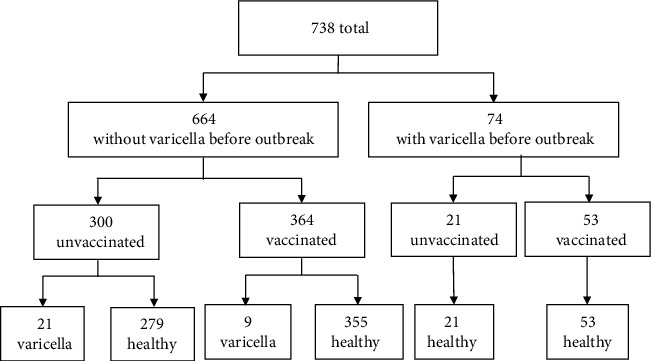
Vaccination and varicella status; a varicella outbreak in a preschool in Suzhou, China (2016) (*n* = 738).

A total of 738 children were attending the preschool during the outbreak. Questionnaires were returned for all 738 children. Overall, vaccination coverage of single-dose varicella vaccine at the outset of the outbreak was 56.5% (417/738). Of the 738 children, 74 (10.03%) with prior history of varicella were excluded for further analysis and none of them developed varicella in the outbreak (Figure 1). Among the remaining 664 children without prior history of varicella, 364 (54.8%) were vaccinated at least 42 months before the outbreak and 300 (45.2%) were unvaccinated. The average age of the 664 children included in the retrospective study was 58 months (range: 42-78), and 53.8% (357/664) was male. Among the 364 children vaccinated, the average age at vaccination was 18 months and the average time since vaccination before the outbreak was 40 months.

### 3.2. Investigation of the Index Case

The index case was a 6-year-old unvaccinated child in top class 6, who was a regular attendee between February 22^nd^ and March 4^th^. He was found in the absence record of the preschool from March 7^th^ to 11^th^, and he had a trail of several skin rashes scabbed over and fell off on March 18^th^ through screening. The peak time of cases cluster in the outbreak was March 17^th^. Based on two weeks of the average incubation period, the index case might appear to be infected with varicella on March 3^rd^ (Thursday). Then, he introduced varicella to top class 6 in the preschool, which was the most affected class during the outbreak, with 25 of 30 varicella children (83.3%) in the preschool.

### 3.3. Outbreak

The outbreak lasted 6 weeks, beginning on March 3. All 30 varicella cases were distributed in 4 adjoining top classes. The mean age of children with varicella was 72 months (range: 66 to 77 months). 21 cases (70%) occurred in unvaccinated children, and 9 cases were breakthrough varicella. The overall attack rate (AR) in the preschool was 4.5% (30/664). No case was found in bottom classes and middle classes. 25 (83.3%) cases occurred in the top class 6 (AR = 62.5%, 25/40), 3 (10.0%) cases occurred in the top class 5 (AR = 7.5%, 3/40), 1 (3.3%) case occurred in the top class 4 (AR = 2.5%, 1/40), and 1 (3.3%) case occurred in the top class 3 (AR = 2.5%, 1/40). The ARs in boys and girls were 3.1% and 6.2%, respectively (*P* = 0.055). Among the 364 single-dose recipients, age at vaccination (<15 months vs. ≥15 months) and time since vaccination before the outbreak (<3 years vs. ≥3 years) were not related to the occurrence of breakthrough varicella in the outbreak (*P* = 1.000 and *P* = 0.063, respectively). The concrete comparisons of children with and without varicella are listed in Table 1.

### 3.4. Outbreak Control Measures

Varicella cases, including breakthrough diseases, were isolated from the preschool until lesions were crusted or faded. The class with the most cases of varicella was suspended for 21 days. Simultaneously, group activities were also suspended and the classrooms, indoor play area, and other public places were disinfected when the outbreak was detected.

### 3.5. Epidemic Curve

**Figure 2:**
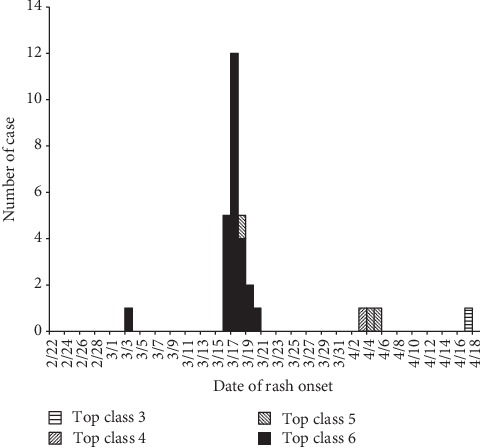
Epidemic curve of a varicella in a preschool in Suzhou, China (2016).

After the index case, 3 generations of cases occurred, with 25 cases in the second generation, 3 cases in the third generation, and 1 case in the fourth generation (Figure 2). The intervals of every generation were approximately 2 weeks.

### 3.6. Vaccination Effectiveness (VE)

**Figure 3:**
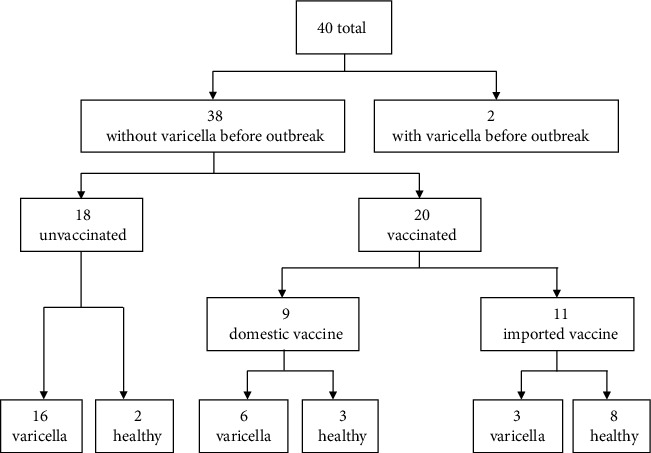
Vaccination and varicella status of top class 6; a varicella outbreak in a preschool in Suzhou, China (2016) (*n* = 40).

Among all the 664 children included in the study, the ARV was 2.5% (9/364) and the ARU was 7.0% (21/300). The difference of ARs in the unvaccinated and vaccinated children was suggested to be statistically significant (*P* < 0.05). VE was 64.7%. For the 40 children of top class 6, 2 children had prior history of varicella and none of them developed varicella. Among the remaining 38 children without prior history of varicella, 18 (47.4%) were unvaccinated and 20 (52.6%) were vaccinated (Figure 3). In calculating the VE of top class 6, the 2 children with prior history of varicella and the index case were excluded. The ARV and ARU were 45.0% (9/20) and 88.2% (15/17), respectively (*P* = 0.014). VE was 49.0%.

## 4. Discussion

The varicella outbreak lasted 6 weeks in the preschool of Suzhou where single-dose varicella vaccine coverage before the outbreak was nearly 54.8%; disease was introduced by an unvaccinated child and occurred highly centralized in a class. The index case has not been found through routine monitoring and had not been isolated from the preschool timely, which ultimately led to the outbreak. This study has confirmed that single-dose varicella vaccination is effective in preventing cases, but moderate single-dose varicella vaccination coverage may not provide sufficiently high population immunity to prevent the outbreak.

Single-dose varicella vaccine has been demonstrated to be highly effective in preventing outset of disease [11–14]. In the United States, attack rates of varicella in school with high and low single-dose varicella vaccine coverage were remarkably different, from 9% to 15% in schools with vaccine coverage levels higher than 95% to 55% in a school with vaccination coverage of just 45% [15–19]. In preschools and schools of China, attack rates were less than 10% in high vaccination coverage while more than 50% in low vaccination coverage during outbreaks reported [20–22]. The efficacy of single-dose varicella vaccine was high in clinical trials, and postlicensure studies confirmed that 80% of VE would need to be reached to prevent varicella and prevent moderate and severe disease [23]. Our investigation reaffirmed the effectiveness of single-dose varicella vaccine, but the VE in the outbreak was low. The VE in the class of the index case was only 49.0%, and the AR of breakthrough case was 64.7%. On the other hand, compared with unvaccinated cases, previously vaccinated cases (breakthrough cases) had a shorter duration of varicella in the outbreak (*P* < 0.05). Our investigation has demonstrated, as have others [24, 25], that single-dose varicella vaccination may have a significant effect on attenuation of disease severity in children.

The manufacturer of the imported single-dose varicella vaccine in the outbreak was GSK, while the domestic vaccines were from Shanghai Biological, Changchun Biological, Changchun Changsheng, and Changchun Baike companies. There were 9 breakthrough varicella cases in the outbreak, who were in top class 6. The difference of the attack rates between domestic vaccine and imported vaccine in top class 6 and the whole preschool was shown to be not statistically significant (*P* > 0.05), suggesting that the imported vaccine might not appear to perform better than the domestic vaccine in the outbreak. Meanwhile, two meta-analyses have demonstrated that the difference in the serum antibody seroconversion incidences among domestic vaccine and imported vaccine or different brands of domestic varicella vaccines in the Chinese children both was shown to be not statistically significant [26, 27]. However, comparing the efficacies of each domestic vaccine independently in the present study was unsuitable to carry out in the present study owing to the small number of sample and many brands of domestic varicella vaccine vaccinated in the preschool. Thus, further studies based on a larger sample study and better design are needed to explore protective efficacy of different domestic varicella vaccines in the future.

In Suzhou, varicella vaccine was not included in national immunization program for children, which is available for private purchase, with a single dose currently recommended for children aged >12 months. In the preschool we investigated, the overall single-dose varicella vaccination coverage was only 56.5%, which was not sufficient to prevent the outbreak, and previous studies also indicated that high single-dose varicella vaccine may not prevent outbreaks. Halloran et al. predicted that 97% of varicella vaccination coverage would need to be reached to prevent outbreaks [28]. Meanwhile, several studies found that 98-100% of vaccination coverage still did not avoid the occurrence of outbreaks [13, 14, 19, 29]. Owing to the above-mentioned reason, the United States changed from a routine one-dose varicella vaccination program to a universal two-dose program for children in 2006 [30]. The adjustment policy was to prevent varicella outbreaks that continued to occur despite high single-dose vaccination coverage since the implementation of the universal two-dose program in the United States; further reductions have been emerged in varicella disease burden and the number of outbreaks [30–32]. Thus, two-dose varicella vaccination should be considered by the policy makers in the future in Suzhou.

Some limitations of the present study should be considered. First, because of the population confined to the children in preschool, we could not require the association between breakthrough disease and five or more years since vaccination in this study. Second, most cases were clinically diagnosed, we did not require laboratory confirmation, and possible misdiagnosis could lead to overestimated or underestimated VE. Third, rashes mistaken for breakthrough disease could have falsely lowered the estimate of VE, although these occur infrequently in early spring. Fourth, less severe breakthrough disease that was not clinically recognized could have resulted in a false elevation of the estimate of VE.

In summary, the present study suggested that single-dose varicella vaccination was effective in preventing disease, but the AR of breakthrough varicella was high and the low vaccination coverage could not prevent the outbreak. Timely detection and effective isolation are key factors in controlling varicella. Improving single-dose vaccination coverage and implementing two-dose vaccination strategy should be recommended to provide excellent protection to prevent varicella in the future in Suzhou.

## Figures and Tables

**Table 1:** Comparison of children with and without varicella in a varicella outbreak in Suzhou, China, 2016.

Variable	Children with varicella (*n* = 30**)**	Children without varicella (*n* = 634)	*P* value
*Gender*			0.055^a^
Male	11 (3.1%)	346 (96.9%)	
Female	19 (6.2%)	288 (93.8%)	
*Age (months)*			
Range	66-77	42-78	
Mean (SD)	72.1 (3.3)	57.8 (10.7)	
*Vaccination status*			0.005^a^
Unvaccinated	21 (7.0%)	279 (93.0)	
Vaccinated	9 (2.5%)	355 (97.5%)	
*Age at vaccination (months)*			1.000^b^
<15 months	3 (2.1%)	142 (97.9%)	
≥15 months	6 (2.7%)	213 (97.3%)	
*Time since vaccination before outbreak*			0.063^b^
<3 years	0 (0.0%)	108 (100%)	
≥3 years	9 (3.5%)	247 (96.5%)	

^a^Pearson's chi-square test. ^b^Fisher's exact test.

## Data Availability

Data used in this study are available through the official website of MICS to registered users.
